# RAC-Multi: Reader Anti-Collision Algorithm for Multichannel Mobile RFID Networks

**DOI:** 10.3390/s100100084

**Published:** 2009-12-24

**Authors:** Kwangcheol Shin, Wonil Song

**Affiliations:** Department of Information & Communications Engineering, Korea Advanced Institute of Science and Technology, 119, Moonji-Ro, Yuseong-Gu, Daejeon, 305-732, Korea; E-Mail: xd3535@kaist.ac.kr

**Keywords:** mobile RFID, reader collision problem, multichannel

## Abstract

At present, RFID is installed on mobile devices such as mobile phones or PDAs and provides a means to obtain information about objects equipped with an RFID tag over a multi-channeled telecommunication networks. To use mobile RFIDs, reader collision problems should be addressed given that readers are continuously moving. Moreover, in a multichannel environment for mobile RFIDs, interference between adjacent channels should be considered. This work first defines a new concept of a reader collision problem between adjacent channels and then suggests a novel reader anti-collision algorithm for RFID readers that use multiple channels. To avoid interference with adjacent channels, the suggested algorithm separates data channels into odd and even numbered channels and allocates odd-numbered channels first to readers. It also sets an unused channel between the control channel and data channels to ensure that control messages and the signal of the adjacent channel experience no interference. Experimental results show that suggested algorithm shows throughput improvements ranging from 29% to 46% for tag identifications compared to the GENTLE reader anti-collision algorithm for multichannel RFID networks.

## 1. Introduction

Radio frequency identification (RFID) technology enables data to be transmitted from a tiny portable device, called a tag, to an RFID reader and processed according to the needs of a particular application. RFID systems have been applied in various areas of industry, such as supply chain management and the retail business. Recently, with the appearance of the ubiquitous computing era, mobile RFID is emerging. In a mobile RFID system, the reader is installed in a mobile device such as a mobile phone, PDA, cart or even a forklift truck [1]. Mobile RFID is different from the current implementations of ordinary RFID; here the readers are mobile and the tags are fixed, instead of the other way around [2]. The emergence of mobile RFID makes the RFID technology applicable by giving companies more chances to interact with their end-users. Customers who have a mobile phone in which an RFID reader is embedded can scan a product or an object to retrieve or transmit information. This can change the way mobile commerce is done, and had implications on electronic shopping, logistics and payments. In other word, various new business models and services will appear as a result of this new technology [3].

However, to use a mobile RFID system, reader collision problems should be addressed because RFID readers are constantly moving. Reader collisions arise when multiple readers are in close proximity and consequently as a result readers interfere with one another. The interference makes reading a tag difficult [4]. With more than one channel, the reader collision problem is complicated due to the interference between adjacent channels. However, studies involving reader collision problems between adjacent channels in mobile RFID networks are not common.

This study is the first work that defines a new reader collision problem between adjacent channels and also proposes a novel reader anti-collision algorithm for multichannel mobile RFID networks. The main idea of proposed algorithm is to separate data channels into odd- and even-numbered channels and to use the odd-numbered channels first instead of randomly selecting a channel from among all available channels. The proposed algorithm also provides one channel of separation between the control channel and data channels to ensure that interference between control messages and the signal of the adjacent channel does not occur. Experimental results show that the proposed reader anti-collision algorithm outperforms the GENTLE algorithm [5] by 29% to 46% according to the number of readers. The GENTLE algorithm is a very recent method that considers the reader collision problem in multichannel mobile RFID networks.

This study is organized as follows: existing reader anti-collision solutions for mobile RFID are reviewed in Section 2, and a new reader collision problem in multi-channeled mobile RFID is explored in Section 3. In Section 4, the proposed algorithm is explained with the mathematical analysis of the algorithm. Experimental results are given in Section 5 and subsequently concluding remarks are given in Section 6.

## 2. Related Works

RFID reader anti-collision algorithms can be divided into two major categories, according to the mobility of readers. To deal with reader collision problem of static readers, there have been various works and these works can be categorized as either FDMA [6,7], TDMA [8,9] and CSMA [10]. To deal with reader collision problem of mobile RFID reader, there are several works so far as follows:

In [11], they proposed an efficient reader anti-collision algorithm using a polling server in dense and dynamic RFID networks with mobile readers. With the assistance of the server, the readers can rapidly decide whether they can work or not without interfering neighbors. They showed that their algorithm makes readers aware of neighbors to minimize reader collisions.

HiQ [12] utilized three basic hierarchical tiers in its control structure: readers, R-servers, and Q-servers. The lowest tier is the RFID readers which they communicate solely when they have been granted a frequency and time slot in communication by a server (R-server) tier. R-servers are allocated frequencies and time slots by the Q-learning servers or Q-servers. Q-learning servers comprise the highest tier in this hierarchical algorithm. Q-servers distribute resources to the servers directly below them in the hierarchy.

These two works [11,12] are utilizing the servers which have global knowledge to solve reader collision problem. However, in many cases, using servers to resolve the problem takes cost and time.

Birari and Lyer [13] suggested a simple Pulse protocol which separates a control channel from a data channel. A reader, which is communicating with tags, periodically broadcasts beacon messages to an extent greater than its interference range through the control channel. The readers that receive these beacon messages do not prompt for communications with the tags; rather, they wait until they no longer receive beacon messages. In [14], an improved pulse protocol-based reader anti-collision algorithm was proposed for reducing reader collisions via slot occupied probability. They showed that their algorithm improves the reading speed, throughput and system efficiency compared with the conventional anti-collision algorithms.

Another study [15] proposed a cooperative, distributed reader collision avoidance algorithm termed DiCa and insisted that DiCa is suitable for energy-efficient wireless mobile network environments in conjunction with RFID, as this algorithm is capable of not only avoiding collisions but also changing the power state autonomously through simple interactions between adjacent readers.

Recently, a study [16] presented a two phase dynamic modulation (TPDM) technique, which consists of regional scheduling and hidden terminal scheduling phases, aims to efficiently perform communications between readers and tags in high density and mobile RFID networks. They insisted that TPDM is a simple mechanism for coordinating simultaneous transmissions among multiple readers and hidden terminals, and that TPDM can prevent reader collisions by using a distributed self-scheduling scheme.

These algorithms [13–16] were developed based on the assumption that there are only two channels, a control channel and a data channel. However, assuming a single data channel is no longer realistic as the international standards [6,7] suggested multi-channel environments for mobile RFID and, as far as we know, GENTLE is the only work that takes consider multi-channel environments for mobile RFID.

GENTLE [5] suggested a distributed CSMA-based mechanism which uses RFID multichannel and beacon messages to mitigate reader collisions. In this scheme, readers also can put tag information in their beacon messages in order to forward information to nearby readers. GENTLE was shown to be superior to previous CSMA-based reader anti-collision techniques such as LBT [10] and Pulse [13]. However, to read tags, GENTLE randomly selects a data channel from among the available data channels and consequently this leads to interference problems between adjacent channels frequently since GENTLE did not consider interference between adjacent channels.

To use mobile RFID in a multichannel environment, interference between adjacent channels should be considered. This study is the first work that defines a new concept of a reader collision problem between adjacent channels and also suggests an efficient reader anti-collision algorithm for RFID readers that use multiple channels.

## 3. New RFID Reader Collision Problem in Multichannel Environments

In this section, the RFID reader collision problems of single channel are briefly reviewed and then a new concept of reader collision problem between adjacent channels in multichannel RFID networks is explored before the suggested algorithm is explained in Section 4.

### 3.1. Reader Collision Problems in a Single Channel

**Figure 1.**
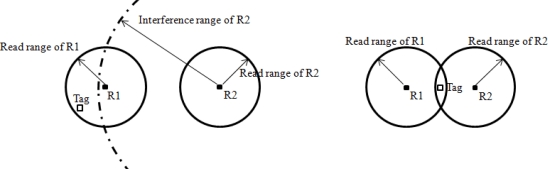
Reader Collision Problems.

In RFID networks that use only one channel, two types of reader collisions can occur: reader-to-reader collisions and multiple reader-to-tag collisions [13]. Reader-to-reader interference arises when a stronger signal from a reader interferes with a weakly reflected signal from a tag, as shown on the left in Figure 1. In this case, the reflected signal of the tag cannot be read by R1 due to the interfering signal of R2. Multiple reader-to-tag interference arises when more than one reader attempt to read the same tag simultaneously (right side of Figure 1). In such a case, the tag cannot be read by either R1 or R2, as the tag cannot decipher any query. The hidden terminal problem in RFID systems, which is also considered as another type of reader collision problem, was discussed in [13], but subsequent research [5] proved that the hidden terminal problem does not occur in actual situations.

All of these problems occur on the assumption that readers use the same channel to read tags. If multiple channels are used to read tags, another reader collision problem can occur.

### 3.2. New Reader Collision Problem between Adjacent Channels

**Figure 2.**
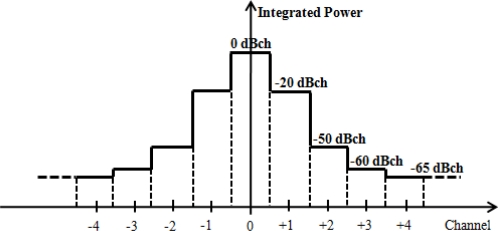
Spectral mask of a channel transmission.

For RFID communications, international standards suggested the use of a frequency between 860 MHz and 960 MHz [6,7]. In Korea, a frequency ranging from 908.5 MHz to 914 MHz with 25 channels of 200 Hz bandwidth each was standardized for mobile RFID networks, as shown in Table 1 and also to minimize adjacent channel interference, the spectral mask of a channel transmission should follow the values shown in Figure 2 [17].

Despite the fact that the interference signal strength of adjacent channels is regulated by −20dBch, as Figure 2 shows, a reader can interfere with the signal of an adjacent channel when the reader tries to read tags.

The width of the interference range of an adjacent channel can be calculated using the RFID path loss model of formula (1), as introduced in [18]. This model shows how far readers should be apart to avoid reader-to-reader collisions, as follows:

(1) $$\text{PL}(\text{dB})=\{\begin{array}{c} 32+25\;\text{log}(d),\;\;\text{if}\;0\leq d<8m\\ \;\;\;23+35\;\text{log}(d),\text{if d}\geq 8m \end{array},\text{where}\;d\;\text{is a distance from a reader}$$

**Figure 3.**
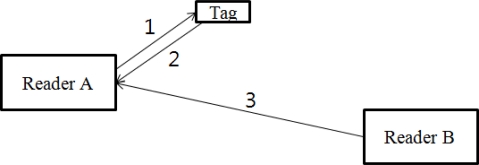
Interference caused by Reader B

If a tag and two readers are located as shown in Figure 3 and the distance between the tag and reader A, which is attempting to read the tag, is 1 meter, the distance between reader A and reader B should be greater than 28.7 meters according to following reasoning: the path loss of reader A to the tag, which is denoted as path 1 in Figure 3, is 32dB according to formula (1). The same amount of pass loss occurs in the reverse path, the tag to the reader (path 2). As 10dB is required to activate the tag [19], the total losses are determined to be 74dB (32 + 32 + 10). To receive the backscattered signal from the tag successfully at reader A, the path loss of the interference signal from reader B to reader A, path 3, should be greater than 74dB. The distance between reader A and B can be calculated as follows:

$$\begin{array}{c} 23+35\text{log}\;(d)>74\\ d>28.7 \end{array}$$

The case above assumes that reader A and B use the same channel. The distance between reader A and B when they use adjacent channels can be calculated as follows; as the signal strength of adjacent channels is reduced by a spectral mask as small as −20dB, as Figure 2 shows.

$$\begin{array}{c} 23+35\text{log}\;(d)>74-20\\ d>7.7 \end{array}$$

**Figure 4.**
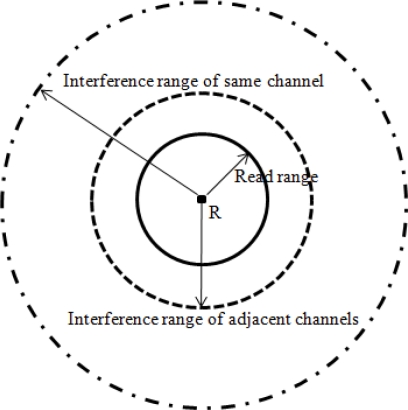
Interference ranges of a reader.

By adopting the above formula, the interference reader-to-reader distances for readers that use same channel and adjacent channels can be calculated, as shown in Table 2. Figure 4 shows a conceptual view of the interference ranges of a reader in a multichannel RFID network.

## 4. Suggested Algorithm for Multichannel Mobile RFIDs

In this section, the proposed algorithm for multichannel mobile RFID networks is explained with a mathematical analysis.

### 4.1. Explanations on the Algorithm

The basic idea of the proposed algorithm involves the use of odd-numbered data channels first since odd-numbered channels have one more channel than that of even-numbered in [17]. If all odd-numbered channels are used, even-numbered channels are then used. Odd- and even-numbered data channels are used separately to minimize interference between adjacent channels by providing one channel of separation.

The channel usage scheme of the proposed algorithm is shown in Table 3. Channel 1 is used as a control channel. It sends beacon messages between readers. Channel 2 is not used given that the signals of channel 2 can interfere with the beacon signal of channel 1. Thus, to ensure that the beacon messages are received without interference, channel 2 remains unused.

The proposed reader anti-collision algorithm for multichannel RFID networks, which considers adjacent channel interference, works as follows. First, when a reader joins the RFID network, it sends a beacon message to other readers to recognize which channels are being used by other readers. It should be noted that if a reader sends a message with signal strength of 30dB, it can communicate with other readers located in an area of nearly 1,000 meters, as the sensitivity to receive a signal by a reader is −101 dB, which is far more sensitive than the tags [19]. This indicates that a reader can communicate with all other readers which are located throughout the entire area of a specific place, such as a supermarket or a library. Once a reader perceives the channels used by other readers, it checks first whether all odd-numbered channels are being used; if odd-numbered channels are available, it randomly selects one of them to use it to read tags. If all of odd-numbered channels are in use, the reader checks for available even-numbered channels and selects one of them randomly. If all odd- and even-numbered channels are in use, the reader then selects randomly one of the channels regardless with either an even or odd number. Once a reader selects a channel, it begins to read tags with that channel using the GENTLE algorithm and counts the number of successful tags reads, denoted as the throughput, during one second. The average throughput of a reader can be calculated using the throughput of each second. In addition, if the current throughput is lower than the average throughput by the amount of a threshold value, the channel is considered to be causing interference with other readers frequently. In this case, the reader changes its channel through executing the channel selection procedure again. The pseudo code of the proposed algorithm is shown in Table 4.

### 4.2. Mathematical Analysis of the Suggested Strategy

As *n* readers are assigned to *k* channels, the probability (*P*) of non-interference between adjacent channels according to the assigning strategies can be calculated as follows. In case of $n>\langle \frac{k}{2}\rangle$, the interferences always happen regardless the strategy of reader assignments.

P(k,n)=0

**Figure 5.**
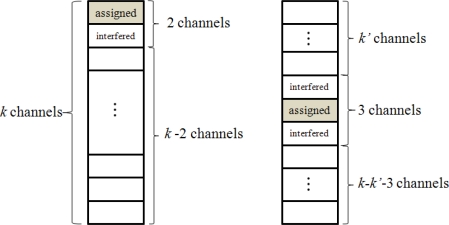
Random channel assignments

In case of $1\leq n\leq \langle \frac{k}{2}\rangle$, if the random reader assignment is applied (Figure 5),

$$\begin{array}{c} P(k,n)=1,\text{where}\;n\leq 1\\ P(k,n)=\frac{2}{k}\times P(k-2,n-1)+\frac{k-2}{k}\times P(k^{\prime },n^{\prime })\times P(k-k^{\prime }-3,n-n^{\prime }-1),\\ \text{where n}>1,\langle \frac{k^{\prime }}{2}\rangle \geq n^{\prime }\;\;\text{and}\;\;n^{\prime }\;\;\text{is the number of readers assiged to}\;k^{\prime }\;\text{channels} \end{array}$$

In case of $1\leq n\leq \langle \frac{k}{2}\rangle$, if the suggested strategy is applied, it never brings channel interference because the suggested strategy assigns a channel, which is one channel away from the used one, to a reader:

P(k,n)=1

This means that the suggested channel assigning strategy clearly has better probability of non-interference between adjacent channels than that of random assignment.

## 5. Experimental Results

To evaluate the proposed algorithm, the RAC-Multi simulator was implemented with C. The details of the simulation model are illustrated in table 5. The maximum output power of the reader is as much as 30 dBm by following the standard for RFID in Korea [17], which indicates that the read range of a reader can extend to about three meters along with −10 dBm to activate tag and −101 dBm of reader sensitivity [19].

If a tag is located three meters away from a reader, the interference range of the same channel is 137.6 meters; in addition, it is 36.9 meters for the adjacent channel, as shown in Table 2. We tested the proposed algorithm in a rectangle space that was 50 meters by 50 meters and in which 100 tags were randomly distributed. The number of readers varied, and was 20 to 60 for sparse, medium and dense cases. The movement of a reader followed a random waypoint mobility model with the speed of 1m/sec., which reflects the movement of a person’s walking, and a reader attempted to read tags 10 times per second. The value for deciding to find a new channel, the threshold value, was set to 0.8, which means that a reader finds a new channel if the current throughput is lower than the average by 20%. Table 6 shows the parameters for the simulation in details. GENTLE algorithm [5] was chosen to be compared with RAC-Multi since GENTLE is the only work, as far as we know, that considers multiple data channels in RFID networks.

**Figure 6.**
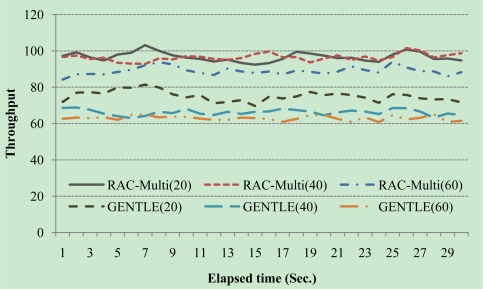
Throughput comparisons.

Figure 6 shows the result of 20, 40, and 60 cases. Here, throughput means that the summation of successful readings of tags per one second by the readers:

$$\text{Throughput}=\sum_{i=1}^{n}\text{successful tag read}(i)$$

As Figure 6 shows, the throughputs of RAC-Multi are better than GENTLE during all of experiment time and Table 7 shows the average throughputs of each algorithm according to the number of readers. The RAC-Multi achieves better results than GENTLE, especially with a medium number of readers.

These results can be inferred that if lots of readers are gathered in small area then the interferences happen frequently and as a result the suggested algorithm and GENTLE come to similar probabilities of adjacent channel interference as explained in section 4.2. And also if small number of readers exists in the area, the gap of throughput is narrowed down since adjacent channel interference does not happen frequently.

## 6. Conclusions

Mobile RFID systems are currently used in various fields of industry. Recently, multichannel systems have been applied in mobile RFID networks. As the use of these new systems increases, adjacent channel interference problems arise. In this work, a new reader collision problem that can occur in multichannel RFID networks is defined, and a novel algorithm that avoids adjacent channel interference is proposed. The proposed algorithm shows better throughput compared to the GENTLE algorithm, which adopts a random channel selection strategy.

## Figures and Tables

**Table 1.** Channel numbers and bandwidths for mobile RFID in Korea.

**Channel No.**	**Channel Frequency**	**Channel No.**	**Channel Frequency**
Protection band	908.50 MHz∼908.75 MHz	14	911.35 MHz∼911.55 MHz
1	908.75 MHz∼908.95 MHz	15	911.55 MHz∼911.75 MHz
2	908.95 MHz∼909.15 MHz	16	911.75 MHz∼911.95 MHz
3	909.15 MHz∼909.35 MHz	17	911.95 MHz∼912.15 MHz
4	909.35 MHz∼909.55 MHz	18	912.15 MHz∼912.35 MHz
5	909.55 MHz∼909.75 MHz	19	912.35 MHz∼912.55 MHz
6	909.75 MHz∼909.95 MHz	20	912.55 MHz∼912.75 MHz
7	909.95 MHz∼910.15 MHz	21	912.75 MHz∼912.95 MHz
8	910.15 MHz∼910.35 MHz	22	912.95 MHz∼913.15 MHz
9	910.35 MHz∼910.55 MHz	23	913.15 MHz∼913.35 MHz
10	910.55 MHz∼910.75 MHz	24	913.35 MHz∼913.55 MHz
11	910.75 MHz∼910.95 MHz	25	913.55 MHz∼913.75 MHz
12	910.95 MHz∼911.15 MHz	Protection band	913.75 MHz∼914.00 MHz
13	911.15 MHz∼911.35 MHz		

**Table 2.** Interference distances according to channel differences.

**Distance of Tag (meters)**	**Total Loss (dB)**	**Interference distance (meters)**	
		**Same channel**	**Adjacent channel**
1	74.0	28.7	7.7
2	89.1	77.1	20.7
3	97.9	137.6	36.9

**Table 3.** Channel usages of the suggested algorithm.

**Channel No.**	**Usage**
1	Control channel
2	Not used
3–25	Data channel

**Table 4.** Pseudo code of the proposed algorithm.

**join()::**
used_channels = beacon message (‘which channel do you use?’, broadcast);
channel = randomly select one of odd numbered channels which is not used (used_channels);
if (channel is null) // all odd numbered channels are being used
channel = randomly select one of even numbered channel which is not used (used_channels);
if (channel is null) // all channels are being used
channel = randomly select channel among data channels;
start_read (channel);

**start_read(channel)::**
total_throughput=0; count=0;
while(reader is in the area)
count++;
// once a reader start to read tags, it follows the GENTLE algorithm
throughput=GENTLE(channel, 1); // throughput during 1 second using GENTLE
total_throughput+=throughput;
average_throughput= total_throughput/count;
if(throughput<threshold×average_throughput)
break while loop;
end while;
if (reader is in the area) join(); // re-selecting a channel to read tags

**Table 5.** Details of the simulated system components

**System Component**	**Power**
Reader RF output power	+30 dBm
Reader/tag antenna gain	0 dB
Power to activate tag	−10 dBm
Reader sensitivity	−101 dBm

**Table 6.** Parameters for the simulation

**Parameter**	**Value**
Simulation range	50 m × 50 m
Time to read one tag	0.1 sec.
Beacon period	0.1 sec.
Simulation time	30 sec.
Mobility model	Random way point mobility model
Movement speed	1 m/sec.
Read range of a reader	3 m
Interference range of the same channel	137.6 m
Interference range of adjacent channels	36.9 m
No. of readers	20, 30, 40, 50, 60
No. of tags	100
No. of channels	25
Threshold to find new channel	0.8
Comparing algorithm	GENTLE

**Table 7.** Average throughputs and improvements

**No. of readers**	**RAC-Multi**	**GENTLE**	**Improvement**
20	96.74	75.14	29%
30	97.82	70.06	40%
40	96.42	66.18	46%
50	95.8	65.74	46%
60	88.94	62.94	41%
